# Antimicrobial Resistance and Biofilm Formation of *Escherichia coli* Isolated from Pig Farms and Surroundings in Bulgaria

**DOI:** 10.3390/microorganisms11081909

**Published:** 2023-07-27

**Authors:** Mila D. Kaleva, Yana Ilieva, Maya Margaritova Zaharieva, Lyudmila Dimitrova, Tanya Chan Kim, Iva Tsvetkova, Yordan Georgiev, Petya Orozova, Krasimir Nedev, Hristo Najdenski

**Affiliations:** 1 Department of Infectious Microbiology, The Stephan Angeloff Institute of Microbiology, Bulgarian Academy of Sciences, 1113 Sofia, Bulgaria; milakalevavet@abv.bg (M.D.K.); illievayana@gmail.com (Y.I.); zaharieva26@yahoo.com (M.M.Z.); lus22@abv.bg (L.D.); tanya_85@abv.bg (T.C.K.); i.likovska@abv.bg (I.T.); y.georgiev001@gmail.com (Y.G.); 2 National Reference Laboratory for Fish, Mollusks and Crustacean Diseases, National Diagnostic Research Veterinary Institute, 1000 Sofia, Bulgaria; petyorozova@gmail.com; 3 Swine Complex (Svinekompleks) Krumovo Gradishte, Boni Holding AD, 1527 Sofia, Bulgaria; k.nedev@boniholding.com

**Keywords:** *Escherichia coli*, pigs, antibiotic resistance, resistance genes, β-lactamase, ESBL, MALDI-TOF-MS, biofilm

## Abstract

*Escherichia coli* (*E. coli*) is a ubiquitous microorganism with pathogenic and saprophytic clones. The objective of this study was to evaluate the presence, virulence, antibiotic resistance and biofilm formation of *E. coli* in three industrial farms in Bulgaria, as well as their adjacent sites related to the utilization of manure (feces, wastewater in a separator, lagoons, means of transport, and soils). The isolation of single bacterial cultures was performed via standard procedures with modifications, and *E. coli* isolates were identified via matrix-assisted laser desorption/ionization time-of-flight mass spectrometry (MALDI-TOF-MS) and polymerase chain reaction (PCR). The disk diffusion method was used to assess antimicrobial resistance, and PCR was used to detect genes for antibiotic resistance (GAR) (*qnr*, *aac*(3), *amp*C, *bla*SHV/*bla*TEM and *erm*) and virulence genes (*stx*, *stx*2all, LT, STa, F4 and *eae*). The protocol of Stepanović was utilized to measure the biofilm formation of the isolates. A total of 84 isolates from different samples (*n* = 53) were identified as *E. coli*. Almost all demonstrated antimicrobial resistance, and most of them demonstrated resistance to multiple antibiotics from different classes. No virulence genes coding the Shiga toxin or enterotoxins or those associated with enteropathogenicity were detected. No GAR from those tested for quinolones, aminoglycosides and macrolides were found. However, all isolates that were resistant to a penicillin-class antibiotic (56) had *β*-lactamase-producing plasmid genes. All of them had *amp*C, and 34 of them had *bla*TEM. A total of 14 isolates formed strongly adherent biofilms. These results in a country where the use of antibiotics for growth promotion and prophylaxis in farms is highly restricted corroborate that the global implemented policy on antibiotics in human medicine and in animal husbandry needs revision.

## 1. Introduction

A famous and widely cited report stated that 10 million people will die each year by 2050 due to antimicrobial resistance (AMR), popularly known as antibiotic resistance [1]. However, scientists, who have devoted more studies to predictive modeling, have emphasized that, fortunately, this is not to be expected with the current rate of increasing mortality due to AMR but will occur under a very specific worst scenario if no action is taken [2]. According to our observations, such misunderstandings are to be expected, because there are not many research articles on that topic, and we rely on reports for citing. However, a recent and also much cited article estimated that, in 2019, there were 1.27 million deaths attributable to bacterial AMR and 4.95 million deaths associated (indirectly attributable) with it [3]. Given these numbers, it is absolutely justified that The World Health Organization recognized AMR as a top-priority health threat [4].

Besides unnecessary prescriptions in medicine, antibiotics are used in animal husbandry to specifically promote the growth and/or health maintenance of livestock. This can lead to the development of drug-resistant bacteria in the gut of animals and is considered to be one of the main reasons for the rapid exacerbation of AMR worldwide [5,6]. That phenomenon limits the therapeutic options for severe livestock infectious diseases. The antimicrobials used for food-producing animals are frequently the same or belong to the same classes as those used in human medicine. In addition, the use of one class of antimicrobial may result in the selection of resistance against another, unrelated class (co-resistance) [7].

Drug-resistant bacteria from the intestines of farm animals are considered a potential source of genes for antibiotic resistance (GAR) that can spread horizontally to zoonotic and other bacteria through the food chain but also through water, manure and direct animal contact, and cause illness [7,8]. On a global scale, about 73% of all antimicrobials sold are used in food animals [9], and that figure is increasing. The domestic pig (*Sus scrofa domesticus*) is one of the main sources of meat for human consumption, as over 40% of all meat consumed in the world comes from this animal species [10]. Some antibiotics, such as fluoroquinolones and tetracyclines, cannot be fully metabolized in the pig intestine, and these residues are found in dust, feces, sewage, soil, surface water and crops. These different groups of antibiotic residues are suitable breeding grounds for resistant bacteria [11,12].

*Escherichia coli* (*E. coli*) represents a major reservoir of antimicrobial resistance genes that can spread horizontally to zoonotic and other bacteria. It is actually intrinsically susceptible to almost all clinically relevant antimicrobial agents, but this bacterial species has a great capacity to accumulate resistance genes, mostly through horizontal gene transfer [13]. *E. coli* also produces extended-spectrum beta lactamase (ESBL), which is also a global health problem. These enzymes are coded by genes such as *bla*TEM, *bla*SHV, *bla*NDM-1, etc. The *bla*TEM gene is expressed with a high value in livestock nurseries, pigs for fattening, and manure. *E. coli* has also been found to have an important role in the spread of *mcr* and/or *tet*(X3)/(X4). These genes collectively mediate resistance in Gram-negative bacteria to drugs such as penicillins, carbapenems, polymyxins and tigecycline, which may lead to a lack of choice of antimicrobials in both human and veterinary medicine [14,15,16,17]. Plasmid-mediated quinolone resistance (PMQR) genes and 16S rRNA methylases are other problematic genetic determinant classes of AMR in *E. coli* [18]. It is recommended to monitor commensal *E. coli* (and enterococci) as a biomarker to monitor AMR in livestock farms, from randomly selected healthy animals, in food and/or in hospitals because their resistance is an indicator of the selective pressure exerted by the use of antimicrobials on intestinal populations of bacteria in food animals [7,19,20,21].

In addition, in 2019, *E. coli* was considered one of the major pathogens responsible for deaths associated with AMR [3]. Shiga toxin-producing *E. coli* (STEC) is the fourth most commonly reported foodborne gastrointestinal infection in humans in the EU [22], which determines the importance of testing for virulence genes.

Although *E. coli* is not as ubiquitous of a pathogen in biofilms found in healthcare as methicillin-resistant *Staphylococcus aureus* and *Pseudomonas aeruginosa* are, it can still cause sepsis [23]. Biofilms are one of the mechanisms of AMR, because they protect the inner bacterial cells as a result of reduced permeability. It is established that bacterial AMR (including resistance to host immune factors) increases up to several hundred times in the biofilm. Persister cells also contribute to reduced sensitivity [24]. For those reasons, we included tests for biofilm formation in our research.

In order to continue our work in the field of antimicrobial resistance in Bulgarian farm swine samples, we tested feces and lagoons in the same way as that in our previous work [25], but this time, we include three farms as well as wastewaters, transport vehicles and soils in addition. The results concerning the antimicrobial susceptibility, GAR and biofilm formation of *E. coli* are presented here.

## 2. Materials and Methods

### 2.1. Swine Farms and Sample Collection

The first pig farm for this research was the same as that in our previous study [25] (near Kostinbrod town). Samples were taken in May 2021. Three fecal samples (FK1–FK3), three samples from lagoons (LK1–LK3), three samples from wastewaters from pigs for fattening (WK1–WK3) and five samples from soils from fields adjacent to the farm/lagoons (SK1–SK5) were collected. Additionally, nine soil samples were taken from soils from fertilized fields adjacent to the farm again in November 2022 (SK6–SK14), adding to a total of 23 samples. Sample FK3 was from young pigs, sample FK2 was from lactating sows, and sample FK3 was from pigs for fattening. Samples LK1, LK2 and LK3 were taken at a depth of 10 cm, 50 cm and 70 cm, respectively. WK1, WK2 and WK3 were taken at a depth of 5, 25 and 40 cm, respectively. SK1, SK2, and SK3 were taken at a distance of 20 cm, 1 m and 4 m from a lagoon, respectively, and at a depth of 16 cm, 20 cm and 26 cm. SK4 and SK5 were taken from a surface layer and at a 16 cm depth, respectively, from a fertilized field.

The second swine farm was in Samovodene village (near Veliko Turnovo town). Four fecal samples (FV1–FV4), four samples from wastewaters drained from a separator and at a depth of 1 m (WV1–WV4), one sample from a lagoon with a depth of 1 m (LV1) and three samples from a 25 cm depth from agriculture fields (with tomatoes) within the limits (vicinity) of the farm (S1–S3) were collected (a total of 12 samples). FV1 and FV2 were from pregnant and lactating sows, respectively. FV3 was from pigs for fattening, and FV4 was from young pigs. WV1, WV2, WV3 and WV4 were from pigs for fattening, young pigs, lactating sows and pregnant sows, respectively.

The third pig farm was in Krumovo Gradisthe village (near Karnobat city). The farm was established before 1980 to meet the needs of meat in the Burgas Province, which is the largest province by area and is privately owned. A total of 2560 sows, about 7000 suckling pigs and 8000 growing pigs are reared on the farm. They are raised until the age of 60 days from birth and are then fattened in another pig-rearing complex—the largest in the Burgas region. At the cultivation premises, the fertilizer mass enters a collective shaft, and from there, it is taken to separators for separating the liquid from the solid phase. The solid phase is separated in concrete basins, and after one month, it can be used for fertilizing. The liquid phase goes into clarifiers, and from there, it goes into “earth fill”-type lagoons, where it stays for about six months. From there, it is spread by a tanker as fertilizer after the harvest period. Six fecal samples (F1–F6), two samples from wastewaters (W1–W2), two samples from lagoons (L1–L2), one sample from a transport vehicle (T1) and seven samples from soils from fields adjacent to the farm (S1–S7) were collected in October 2022 (a total of 18 samples). Samples F1 and F2 were from the feces of lactating sows, samples F3 and F4 were from suckling piglets, and samples F5 and F6 were from young pigs. The samples from the wastewaters were a liquid fraction from the border, with a thicker fraction (W1) and a fresh hard sample from a separator (W2). Lagoon samples were from the surface layer at 5 cm (L1) and from the deep layer at 25 cm (L2). T1 was a hard dry sample from a tractor. Soil samples were from different depths and 3 different fields: S1 was taken at 10 cm from field 1; S2–S4 were taken at 10, 30 and 50 cm, respectively, from field 2; and S5–S7 were taken at the same depths from field 3. All samples were collected according to ISO 5667-3:2018 with the permission of the farm owners.

The farms comprise western, eastern and central Bulgaria, and the total number of samples was 53 (*n* = 53).

### 2.2. Isolation of Single Bacterial Cultures

Single colonies, suspected to consist of *E. coli*, were isolated according to ISO 16654:2001/Amd 1:2017 with some modifications. The enriched samples were cultured on HiCrome™ Chromogenic Coliform Agar (CCA) (M1991I) or Endo agar (M029) (HiMedia, Mumbai, India). Because this study was part of research that included culturing in the search for other bacterial genera, single colonies, suspected of having *Salmonella* spp., were isolated according to ISO 65791:2017. Enriched samples were cultured on XLD agar (M031, HiMedia, Mumbai, India).

For positive controls, we used *E. coli* ATCC 35218 (American Type Cell Culture Collection, Manassas, VA, USA), as well as *E. coli* O:157 and *E. coli* 41 (Collection of the Stephan Angeloff Institute of Microbiology). All isolated colonies were morphologically characterized with the automatic HD colony counter Scan 1200 (INTERSCIENCE, Saint-Nom-la-Bretèche, France).

### 2.3. Identification of E. coli via Spectral Methods (Matrix-Assisted Laser Desorption/Ionization Time-of-Flight Mass Spectrometry) (MALDI-TOF-MS)

All isolated colonies from Endo and XLD agar were identified via MALDI-TOF mass spectrometer (Bruker Daltonics, Billerica, MA, USA). The essence of the technique is the identification of microorganisms by mapping their unique protein pattern. A small amount of an overnight bacterial mass with a density of 10^4^ to 10^6^ CFU/mL was mixed with 1 μL of a matrix solution—*α*-cyano-4-hydroxycinnamic acid (HCCA)—and was placed on the corresponding well of the matrix. The mixture thus made was allowed to dry and was later loaded into the apparatus. Mass spectrometry occurred under constant high vacuum values, and each sample was exposed to short pulses of laser rays (acceleration voltage of 20 kV, mass range of 2.6–20 kDa, laser frequency of 60 Hz and pulsed ion extraction delay of 170 ns). With the energy created from the laser ray, ribosomal proteins were ionized. The molecular fingerprints were comparted with a reference database for ID using the MALDI Biotyper software (Bruker Daltonics, Billerica, MA, USA). The strains identified as *E. coli* were used for further analysis.

### 2.4. Isolation of DNA of the Bacterial Colonies of E. coli

The strains confirmed for *E. coli* via MALDI-TOF were recultured, and the total DNA was extracted from single colonies with either the GeneMATRIX Tissue & Bacterial DNA Purification Kit (E3551, EURx Ltd., Gdańsk, Poland) or the GenElute Bacterial Genomic DNA Kit (Sigma-Aldrich, St. Louis, MO, USA) or through crude lysate preparation. The lysates were made by dissolving one bacterial colony in 100 µL of lysis buffer of 0.05 M NaOH and 0.125% sodium dodecyl sulfate (final concentrations), and samples were incubated for 17 min at 90 °C. The DNA concentration and purity were determined with NanoDrop Lite (Thermo Fisher Scientific Inc., Waltham, MA, USA).

### 2.5. Polymerase Chain Reaction (PCR) Analysis

The extracted DNA from the isolated *E. coli* strains was subjected to conventional and multiplex PCR with the following:

- Gene-specific primers for *E. coli* (genes *uidA*, coding *β*-glucuronidase and *yccT*, coding a conserved protein with an unknown function);
- Primers linked to virulence genes—Shiga toxin (verotoxin)-producing (STEC/VTEC) (*stx* and *stx*2all), enterotoxigenic (ETEC) (LT, STa, and F4) and enteropathogenic (EPEC) (*eae*);
- Primers linked to genes for antibiotic resistance (GAR)—quinolones (*qnr*), aminoglycosides (*aac*(3)), β-lactamase-producing plasmid genes (*amp*C and *bla*SHV/*bla*TEM) and macrolides (*erm*) (Table 1);.
- *Bla*SHV/*bla*TEM codes ESBL, and *amp*C codes AmpC beta-lactamase.

For PCR amplification, we used the Color perpetual Taq PCR Master Mix (2×) protocol (E2745, EURx Ltd., Gdańsk, Poland) optimized in our laboratory, as follows: 1 cycle of initial denaturation running at 95 °C for 5 min; a total of 30 cycles of denaturation (at 94 °C for 30 s), annealing (depending on the melting temperature of the primer for 30 s) and extension (at 72 °C for 1 min); and 1 cycle of final extension (at 72 °C for 7 min) and cooling (at 4 °C). Where lysates were used, Tween-20 and gelatine were added to the reaction mix to final concentrations of 0.5% and 0.01%, respectively. The PCR products were visualized in 1.5 or 2% agarose gels. For positive controls, we used the following strains: *E. coli* ATCC 35218 for the detection of *E. coli* strains (*uidA* and *yccT*), *E. coli* O:157 containing LT and *E. coli* 41 (Collection of the Stephan Angeloff Institute of Microbiology) for the detection of *eae* genes. For the other different *E. coli*, strains from the Collection of the Stephan Angeloff Institute of Microbiology were used.

**Table 1:** List of primers with their sequences and temperature of melting (annealing) (Tm).

Primers	Sequences	Tm	Amplicon	Reference
*E. coli uidA* F	5′-AAA ACG GCA AGA AAA AGC AG-3′	55 °C	147 bp ^1^	[26]
*E. coli uidA* R	5′-ACG CGT GGT TAC AGT CTT GCG-3′			
*E. coli yccT* F	5′-GCA TCG TGA CCA CCT TGA-3′	56 °C	59 bp	[27]
*E. coli yccT* R	5′-CAG CGT GGT GGC AAA A-3′			
*stx*1-1 F	5′-TTA GAC TTC TCG ACT GCA AAG-3′	60 °C	531 bp	[28]
*stx*1-1 R	5′-TGT TGT ACG AAA TCC CCT CTG-3′			
*stx*2all F	5′-TTA TAT CTG CGC CGG GTC TG-3′	60 °C	327 bp	[28]
*stx*2all R	5′-AGA CGA AGA TGG TCA AAA CG-3′			
LT F	5′-TTA CGG CGT TAC TAT CCT CTC TA-3′	60 °C	275 bp	[28]
LT R	5′-GGT CTC GGT CAG ATA TGT GAT TC-3′			
STa F	5′-TCC CCT CTT TTA GTC AGT CAA CTG-3′	60 °C	163 bp	[28]
STa R	5′-GCA CAG GCA GGA TTA CAA CAA AGT-3′			
F4 F	5′-ATC GGT GGT AGT ATC ACT GC-3′	60 °C	601 bp	[28]
F4 R	5′-AAC CTG CGA CGT CAA CAA GA-3′			
*eae* (Intimin) F	5′-CAT TAT GGA ACG GCA GAG GT-3′	60 °C	791 bp	[28]
*eae* (Intimin) R	5′-ATC TTC TGC GTA CTG CGT TCA-3′			
*qnr*A F	5′-GGG TAT GGA TAT TAT TGA TAA AG-3′	50 °C	670 bp	[29]
*qnr*A R	5′-CTA ATC CGG CAG CAC TAT TTA-3′			
*qnr*B F	5′-GAT CGT GAA AGC CAG AAA GG-3′	54 °C	469 bp	[30]
*qnr*B R	5′-ACG ATG CCT GGT AGT TGT CC-3′			
*aac*(3)-IV F	5′-CTT CAG GAT GGC AAG TTG GT-3′	55 °C	286 bp	[31]
*aac*(3)-IV R	5′-TCA TCT CGT TCT CCG CTC AT-3′			
*bla*SHV F	5′-TCG CCT GTG TAT TAT CTC CC-3′	58 °C	768 bp	[32]
*bla*SHV R	5′-CGC AGA TAA ATC ACC ACA ATG-3′			
*bla*TEM F	5′-TCG GGG AAA TGT GCG CG-3′	55 °C	972 bp	[33]
*bla*TEM R	5′-TGC TTA ATC AGT GAG GCA CC-3′			
*amp*C F	5′-AAT GGG TTT TCT ACG GTC TG-3′	58 °C	191 bp	[34]
*amp*C R	5′-GGG CAG CAA ATG TGG AGC AA-3′			
*erm*B F	5′-GAA AAA GTA CTC AAC CAA ATA-3′	45 °C	639 bp	[35]
*erm*B R	5′-AAT TTA AGT ACC GTT AC-3′			

^1^ Base pairs.

### 2.6. Disk Diffusion Method

Antimicrobial susceptibility testing was performed via a standard disk diffusion method, also known as the Kirby–Bauer method, according to the protocols of the CLSI [36]. We again used antibiotics applicable to the treatment of patients, namely meropenem (10 µg, MEM10C Oxoid ltd, Basingstoke, Hampshire, UK), ampicillin (10 µg, SD002-1PK), amoxycillin (25 µg, SD129-1PK), amoxycillin/clavulanic acid (20/10 µg, AUG30C), carbenicillin (100 µg, SD004-1PK), cefamandole (30 µg, SD200-1PK), erythromycin (15 µg, SD013-1PK), streptomycin (10 µg, SD031-1PK), tetracycline (30 µg, SD037-1PK), doxycycline hydrochloride (30 µg, SD012-1PK), chloramphenicol (30 µg, SD006-1PK), nalidixic acid (30 µg, SD021-1PK), ciprofloxacin (5 µg, SD060-1PK), pefloxacin (5 µg, SD070-1PK) and co-trimoxazole (25 µg, SD010-1PK) from HiMedia, India. The results were evaluated according to the cut-off breakpoint values of EUCAST version 12.0, 2022 [37], CLSI, 31st edition [36] and the Manual of BBL Products and Laboratory Procedures [38]. Breakpoint values of erythromycin for other bacterial species were taken for *E. coli*.

### 2.7. Test for Biofilm Formation

We used the protocol of Stepanović et al. [39] with small modifications, as described in Dimitrova et al. [25]. The biofilms were photodocumented with a microscopic configuration Nikon Eclipse-Ci-L (Nikon Instruments Europe BV, Amstelveen, The Netherlands), and the optical density (OD) was measured at 570 nm by using an ELISA reader ELx800 (BioTek Instruments, Winooski, VT, USA). The classification of Christensen et al. (Table 2) was used again to determine the adherence potential [40].

## 3. Results

### 3.1. Isolation of Single Bacterial Cultures

Selected colonies from CCA, Endo or XLD agar were used for the spectral identification of the bacterial species. Endo agar is recommended for the confirmation of suspected members of the coliform group. *E. coli* are expected to have a metallic sheen on this agar. As XLD agar is a selective medium for *Salmonella* spp., no colonies were suspected to be *E. coli*.

### 3.2. Identification by MALDI-TOF-MS and PCR

A total of 85 colonies from different samples were identified as *E. coli* via MALDI-TOF-MS. Later, 84 of them were confirmed with PCR. Isolates that were positive for either the *uidA* or the *yccT* gene were accepted as *E. coli*. Some colonies that did not have a metallic sheen on Endo and that were not suspected for *E. coli* turned out to be this bacterial species (F4.2 and T1.1), and not all colonies that had a metallic sheen on this agar turned out to be this species. Generally, not all strains of a species isolated with a certain selective nutrient medium have all the expected typical morphological characteristics; therefore, this is not a new phenomenon.

It is interesting that six of the identified colonies were previously suspected to be *Salmonella* spp., as they were isolated from XLD agar (W2.4, T1.2, T1.3, S6.1, S6.2 and S7.2). Moreover, when recultured on Endo agar, two of them had a metallic sheen (W2.4 and T1.3), but the rest of them did not.

### 3.3. Antibiotic Resistance from the Disk Diffusion Method

Although erythromycin is not used for *E coli*, we tested it, because there is the potential horizontal transfer of erythromycin GAR to other bacterial species. As can be seen from Table 3, Table 4, Table 5, Table 6, Table 7 and Table 8, all isolates, except six (WV1.7, WV2.1, WV2.2, WV2.6, SV3.2 and SV3.4), had resistance to at least one agent, and many of them had resistance to multiple antibiotics. The resistance varied in wide ranges—from 0% for cephalosporins to 81% for tetracyclines and other agents. *E. coli* was isolated from all types of samples. Our results show that almost all isolates had resistance to multiple antibiotic agents, in line with the global tendency of increases in AMR, including in farm animals [5,6]. The antibiotic class that was associated with the greatest developed resistance was tetracycline (81%), followed by penicillins (56%). The percentage of chloramphenicol resistance was very high in the Karnobat farm and much lower in the other two, averaging 42.9%. The resistance to aminoglycoside was 39.3%. The resistance to trimethoprim/sulfamethoxazole followed the same pattern as that of the other unsorted agent, chloramphenicol, averaging 27.4%. Fluoroquinolones, on the contrary, showed much lower resistance in the Karnobat farm, in comparison to the others, and the average was 20.2%. Resistance to the class of macrolides was low (6%), and resistance to the class of carbapenems and cefamandole was absent.

Multidrug resistance (MDR), defined as resistance to three or more antimicrobial classes of the panel tested, was found for 25 isolates (29.8%).

Although there was a variation in the patterns between farms, fecal samples were resistant to the greatest number of antibiotics (e.g., tetracyclines, penicillins and streptomycin). Likely, the fecal bacteria were subjected to more selective pressure due to the direct consumption of antibiotics, and/or the environmental factors could play a role in losing GAR in some other environments. The fewest *E. coli* were isolated from lagoons and soils, and they had resistance to fewer antibiotics. However, some of them still showed consistent patterns, such as resistance to tetracyclines or, in the case of Karnobat, to chloramphenicol in addition.

### 3.4. Detection of Antibiotic Resistance Genes

**Figure 1:**
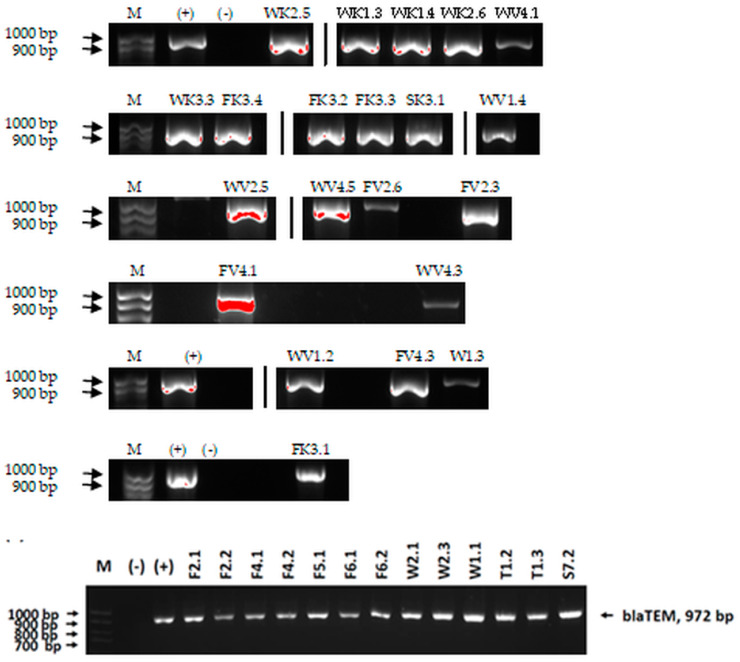
Gel electrophoresis for *bla*TEM *β*-lactam resistant gene. The gel electrophoresis from the farm near Karnobat is confirmative. Black lines designate non-adjacent samples. Legend: M, marker; (+), positive control; (−), negative control.

We sought GAR for a certain antibiotic class only in the isolates that showed resistance to an antibiotic from this class (including to antibiotics not presented in this study). The results (Figure 1) show that, from the group of GAR in this study, the isolates were positive only for β-lactamase-producing genes. They were *amp*C and *bla*TEM. Out of 56 tested isolates, all the samples had the *amp*C GAR, and there were 34 isolates that were positive for *bla*TEM. These results corroborate that ESBL and AmpC β-lactamase production are important resistance mechanisms in members of the Enterobacteriaceae family [18].

### 3.5. Detection of Virulence Genes

No virulence genes from the panel STEC/VTEC (*stx* and *stx*2all), ETEC (LT, STa, and F4) and EPEC (*eae*) were detected among the isolates.

### 3.6. Test for Biofilm Formation

Strongly adherent *E. coli* (14 isolates) was found among all types of samples except the transport vehicle ones (Table 9, Table 10, Table 11 and Table 12). Moderately adherent *E. coli* was present among all types of samples. The Karnobat farm had the most strongly adherent isolates (8), whereas the Veliko Turnovo farm had the least adherent ones (2). Apart from that, there was no correlation in concern to the type of sample and the farm.

## 4. Discussion

The use of antibiotics as growth promoters and for prophylaxis in farms is a highly debated issue. However, there is enough evidence not only for the spread of AMR from livestock, such as swine, to humans (pig feces and wastewater are one of the hotspots for the spread and circulation of AMR and GAR) but also for genetic similarity (clonal types) between resistant bacteria in animals and in humans, some of which are given in a comprehensive review by Sirichokchatchawan et al., 2021 [41]. For example, four sequence types of ESBL producing *E. coli* shared between humans and pigs were found in Thailand [41]. Plasmid (sub)types and, again, ESBL genes such as *bla*_CTX-M-1_ were found to be shared between Dutch pigs and pig farmers [42]. Therefore, the general agreement among policy makers and society is that the disadvantage of creating bacterial AMR outweighs the benefits of antibiotics. Therefore, even though subclinical antibiotic concentrations not only promote growth but also reduce animal morbidity and mortality, numerous bans or restrictions for antibiotic feed additives have been adopted throughout the world [41,43]. The dilemma is excellently described in the work by Chattopadhyay, 2014 [43].

The legislation in Bulgaria is strict, and since approximately the year 2000, antibiotics in animal husbandry have been allowed only as therapeutics under veterinary supervision and with the demand of reporting it to the competent authorities. In poultry farms, most antibiotics are banned even for therapy. An exception in swine farms is the prophylactic use of colistin against post weaning diarrhea.

Nevertheless, in 2010–2016, even newborn suckling pigs still carried as part of their normal intestinal microflora coliforms that had resistance to most tested antibiotic classes, especially to tetracycline and ampicillin. Lactating sows and young pigs had high resistance to streptomycin. Moreover, despite legislative bans, if we compare our results with those from previous studies of swine farms in Bulgaria, an increase in AMR is observed. In the period of 2010–2016, resistance toward tetracycline, ampicillin and streptomycin (approximately 70%, 60% and 65%, respectively) doubled in comparison to the period of 2000–2004 [44]. After a transient decrease in 2020 [25], resistance to tetracycline rose even more to 77.8% and to 81% for the drug class as a whole in our study. Resistance to pefloxacin, carbenicillin and chloramphenicol rose slightly, and there was not a clear correlation regarding the other tested agents [25,44,45].

It is interesting that *bla*TEM was relatively rare in the study of the period of 2010–2016 [44], whereas in this work, *bla*TEM was a very prevalent gene with 34 positive samples from 56 tested. This marks an increase from our last time tested in 2020 [25]. *Amp*C was the only other GAR detected by us with all samples positive out of 56 tested. Last time, it had a similar pattern, because the most numerous positive samples were for that gene [25].

Indeed, there is decreased resistance for some agents, but it still remains relatively high. For example, ampicillin values showed growth in 2021 to 75% but have now decreased to 53.6%. Similarly, streptomycin resistance decreased in 2020 (12.5%) [25] and in this study (39.3%) but still remains high. Amoxicillin resistance decreased in comparison with the period of 2012–2020 [25,45], from 75% to 52.4%. There is very low resistance in farm pigs to third-generation cephalosporins [44] and even to the second-generation agent cefamandole in 2020 [25] and in our study.

As a summary for Bulgarian farm swine, there is high resistance of resident and pathogenic strains to tetracyclines, penicillins and aminoglycosides. Therefore, the high prevalence and increase in AMR in farms with highly restricted antibiotic use (and only for therapy) could indicate residual AMR from past times, the overuse of antibiotics for therapy in farms and/or the high circulation of AMR in the environment due to the high use of antibiotics by humans. This is enhanced by travel and transport in our global society, raising the spread of resistant strains.

The rate of antibiotic resistance differs considerably from country to country globally, depending upon the amount of usage. In the European Union (EU), the lowest levels of AMR *E. coli* isolates have been found in countries where lower antimicrobial usage is practiced, such as in Norway, Sweden and Finland, whereas countries with high levels of use, such as Spain, Portugal and Belgium, have relatively higher levels of AMR *E. coli* [46]. For instance, a clear spatial pattern was detected for tetracycline resistance, with high resistance levels reported for southern and western European countries and much lower levels reported for northern and eastern countries in 2004–2007 [7].

In 2019, some antimicrobial classes were assigned the highest priority, with critically important antimicrobials for human medicine only being available for food animals through veterinary prescription [41]. These are fluoroquinolones, third- and fourth-generation cephalosporins, carbapenems, macrolides and polymyxins (colistin). An increase in resistance to these antibiotics in *E. coli* in animals may indicate a general resistance trend of concern among Gram-negative bacteria originating from the animal reservoir. Carbapenems may not be used in food-producing animals in the present, but it is alarming that resistance genes have been found in pigs, chickens and other livestock [47].

The monitoring and reporting of resistance data from indicator organisms (commensal *E. coli* and enterococci) to the European Food Safety Authority (EFSA) is voluntary. EFSA reports show that, for a timeframe of 2004–2020, resistance to nalidixic acid is, in general, low, as it was in our studies. However, in the past (2004–2007), large variability was observed in the reported ciprofloxacin resistance median levels (4–24%), with the highest occurrence of 74% reported by Estonia in 2007. In Denmark, legal restrictions have been in place for the use of fluoroquinolones in food animals since 2002, and as a consequence, ciprofloxacin resistance decreased from 3% in 2004 to 0% in 2007 [7]. Wider legal restrictions likely led to the low overall resistance to ciprofloxacin now (median approximately 5%) [48]. It is noteworthy that resistance to third-generation cephalosporins is very low, usually below 1%, in European as well as in local Bulgarian pig farms; therefore, resistance to that agent is still not a threat in animal husbandry, unlike in clinical settings [7,48]. Regarding human infections with *E. coli*, the European Antimicrobial Resistance Surveillance Network (EARS-NET) reported in 2017 that the highest population-weighted mean resistance percentage in the European Union and the European Economic Area for *E. coli* that causes serious infections was to aminopenicillins (58.7%), followed by fluoroquinolones (25.7%), third-generation cephalosporins (14.9%) and aminoglycosides (11.4%). In 2017, resistance to carbapenems remained rare in *E. coli* [49].

In 2019–2020, reports of farm swine from 30 countries showed that high or very high resistance to ampicillin, sulfamethoxazole, trimethoprim and tetracycline was the most common resistance trait observed. MDR was observed in 34.2% (versus 29.8% in our study) of commensal *E. coli* isolates from pigs. A wide variety of resistance patterns were observed, but mostly to tetracycline, ampicillin, sulfamethoxazole and trimethoprim, often in combination with other substances but rarely with quinolones. About half of the porcine MDR isolates (52.3%) were resistant to all these antimicrobials. Meropenem resistance was not detected in any isolate of indicator *E. coli*, in line with the results from Bulgaria [48].

Complete susceptibility to 14 antimicrobials tested was higher than that in our study without a statistically significant difference between countries. The aminoglycoside gentamicin had low median levels of resistance through 2004–2020, unlike in Bulgaria. The median levels of chloramphenicol resistance for all reporting countries were moderate in pigs, unlike the high resistance in Bulgaria [48].

There were positive trends (decreases in the level of resistance) in several countries that were possibly due to the documented overall decline in sales of antimicrobials. Most notably, tetracycline resistance has decreased in 15 countries and increased in only two. In 13 countries, there were only decreasing trends, notably in the Netherlands for four agents and in Cyprus for three agents [48]. Indeed, countries such as Denmark and the Netherlands, which both have had massive swine production in recent years, have achieved tremendous reductions in antimicrobial usage while sustaining peak production. Comparable results have been accomplished in Belgium, France, Sweden and the United Kingdom [7,46,48,50]. In contrast, in six countries, there were only increasing trends, and there were increasing trends in Belgium (despite the reduction in antibiotic use), Poland and Romania for three antimicrobials [48].

In reference to the genetic profile, unlike our results, presumptive ESBL producers were more common than AmpC-producers, and isolates with a combined phenotype (ESBL + AmpC) were uncommon. The occurrence of presumptive ESBL, AmpC or ESBL + AmpC producers in commensal *E. coli* was 1.3% in fattening pigs. In pork, the prevalence of presumptive *E. coli* ESBL and/or AmpC-producers in meat was less variable, ranging from 0% (Finland and the Netherlands) to 24.4% (Portugal) [48].

It is interesting that, in the period of 2004–2007, AMR to commonly used antimicrobials was higher in porcine *E. coli* than that in isolates from chickens and cattle, and in most cases, the countries that reported a high occurrence of AMR in *E. coli* from chickens also had a high occurrence of resistance in *E. coli* from pigs [7]. This was likely due to the fact that the global average annual consumption of antibiotics for swine (172 mg/kg) is greater than that for cattle (45 mg/kg) and chickens (148 mg/kg) [51].

As a summary for the EU, the EFSA Animal Health and Welfare Panel (2021) revealed clinical swine *E. coli* isolates with a high proportion of resistance to numerous antibiotics, with a prevalence from 63% to 70% (to aminopenicillins, sulfonamides and tetracycline). However, lower rates of resistance to clinically critical antibiotics (fluoroquinolones and third-generation cephalosporins) were detected [50]. Likely, the latter was the first fruit of the recent Regulation (EU) 2019/61 on Veterinary Medicines and Regulation (EU) 2019/4 on Medicated Feed, stating that antibiotics shall not be applied routinely, nor for prophylaxis, unless in exceptional cases. They should only be applied for metaphylaxis (treatment of animals without signs of disease, which are in close contact with animals that do have evidence of infectious disease) when the risk of spreading infection is very high and there are no other options, as Barros et al., 2023, described [18]. Differences in resistance between countries might reflect the dissemination of certain *E. coli* types within animal populations and/or differences in the consumption of antimicrobials in animals among countries [7,48].

The recommendations that could make the use of antibiotics as a feed additive unnecessary are several, including improved hygiene and vaccines (although their efficacy varies considerably), biosecurity (measures taken to prevent disease introduction, such as monitoring animals or plant materials that enter the property, as well as sources of water and feed). Numerous alternatives to antibiotics exist—prebiotics, probiotics, phytogenic substances, bacteriophages, etc. However, they have their limits for therapy and are mostly used for prophylaxis [18]. The development of alternatives for the clinical management of the infections of livestock appears to be the need of the hour [43]. There is a need to establish national surveillance programs and effective policies, particularly in certain world regions, to curtail the threat of the evolution of resistant isolates in swine or other livestock production [33,52].

It is clear that more unhygienic farms (e.g., in the developing world) demand more antibiotics in their feed. However, antimicrobials as a feed additive reduce morbidity and mortality in most hygienic farms in the developed world [43]. Whether farm animals are exposed to more infectious agents than animals in their natural habitat is a question beyond the scope of this work. However, it is clear that wildlife has access to more open ventilated spaces and disinfecting sun beams. Therefore, our hypothesis is that, as the number of natural conditions that livestock lives in increases, fewer antibiotics are needed for growth promotion.

Biofilm formation by foodborne pathogens is a serious threat to food safety and public health [53]. As a foodborne pathogen, *E. coli* can adhere to and form biofilms on most materials and under almost all environmental conditions in food production plants [54]. In this context, this is of importance for irrigation installations and meat processing plants, given the fact that *E. coli* can survive for months on dry surfaces [55]. Viable pathogens in detached biofilms from contact surfaces can lead to cross-contamination. Environmental biofilms are most often composed of multispecies microorganisms, and mixed biofilm formation can enhance the sanitizer tolerance of foodborne pathogens. *E. coli* is capable of forming biofilms with other bacterial species, and that could enhance its pathogenic clones’ survival in the biofilm community. Biofilm formation in commercial meat plants may be a source of product contamination with no identifiable cause [53].

Even after the cleaning and disinfection processes, the biofilm could still persist. For example, in nursery units in a pig facility after an extensive washing protocol that included disinfection and being kept empty for two weeks, for *E. coli* and fecal coliforms, reductions of 41% and 51% were observed, respectively; however, they were still found on floors, drinking nipples and feeding troughs [55].

Although we did not find pathogenic clones in the present research, the ability to form strongly adherent biofilms for approximately 17% of the isolated *E. coli* commensals that were resistant to commonly used antimicrobials and that were MDR strains is alarming, because a detached biofilm can lead to the spread of the AMR to the environment and bacteria in humans through horizontal gene transfer.

As future directions for our research, colistin resistance could be studied because of its prophylactic use against post weaning diarrhea.

## 5. Conclusions

Although some antimicrobial agents show a lower level of resistance in Bulgarian porcine farms in comparison to European ones (e.g., ciprofloxacin), the higher prevalence of resistance to aminoglycosides in Bulgaria is alarming, and so is the higher level of resistance to tetracyclines, because it is already high in the European Union. Antibiotic stewardship is the effort to improve how antibiotics are prescribed by clinicians and used by patients. In terms of antimicrobial stewardship programs, national action plans in many countries cover both human and animal health sectors [41]. Legal restrictions lead to positive trends, but our study is an example of the relatively high prevalence and increase in AMR in farms with banned antibiotics as feed additives and prophylaxis. This is likely the result of their overuse for therapy in farms and/or the high circulation of AMR in the environment due to the high usage of antibiotics by humans. Antimicrobial utilization should be more correctly structured as a dosage and course of treatment.

## Figures and Tables

**Table 2:** Correlation between the optical density of samples and bacterial adherence [40].

Formula	Adherence
OD_sample_ ≤ OD_blank_	non-adherent
OD_blank_ < OD_sample_ ≤ 2 × OD_blank_	weakly adherent
2 × OD_blank_ < OD_sample_ ≤ 4 × OD_blank_	moderately adherent
4 × OD_blank_ < OD_sample_	strongly adherent

**Table 3:** Antibiotic resistances from the disk diffusion method of the isolated *E. coli* strains from the farm near Kostinbrod (FK3.1–SK3.1).

Drug Class	Antibiotic/Strain	FK3.1	FK3.2	FK3.3	FK3.4	FK3.5	WK1.3	WK1.4	WK2.5	WK2.6	WK3.3	LK1.1	LK1.3	LK1.6	LK2.1	LK2.2	LK3.1	LK3.4	SK3.1
Tetracyclines	Tetracycline	**R**	**R**	**R**	**R**	**R**	**R**	**R**	**R**	I	I	**R**	I	**R**	**R**	**R**	**R**	**R**	**R**
	Doxycycline hydrochloride	**R**	**R**	**R**	I	**R**	I	I	**R**	**R**	I	I	S	S	I	S	S	**R**	**R**
Macrolides	Erythromycin	I	I	I	I	I	I	I	I	I	I	I	I	I	S	I	I	I	I
Cephalosporins	Cefamandole	S	S	S	S	S	S	S	S	S	S	S	S	S	S	S	S	S	S
Fluoroquinolones	Nalidixic acid	S	S	S	S	S	**R**	**R**	**R**	**R**	S	S	S	S	S	S	S	S	S
	Pefloxacin	S	S	S	S	I	**R**	**R**	**R**	**R**	S	I	**R**	I	**R**	**R**	I	**R**	S
	Ciprofloxacin	S	S	S	S	S	**R**	I	I	I	S	S	S	S	S	S	S	S	S
Penicillins	Ampicillin	**R**	**R**	**R**	**R**	**R**	**R**	**R**	**R**	**R**	S	S	S	S	S	S	S	S	**R**
	Amoxicillin	**R**	**R**	**R**	**R**	**R**	**R**	**R**	**R**	**R**	**R**	S	S	S	S	S	S	S	**R**
	Amoxicillin/clavulanic acid	**R**	**R**	**R**	**R**	**R**	**R**	**R**	**R**	**R**	S	S	S	S	S	S	S	S	**R**
	Carbenicillin	I	S	S	S	I	**R**	I	**R**	I	S	S	S	S	S	S	S	S	I
Carbapenems	Meropenem	I	S	S	S	I	S	S	I	S	S	S	S	S	I	S	S	S	I
Aminoglycosides	Streptomycin	**R**	**R**	**R**	**R**	**R**	**R**	**R**	**R**	**R**	S	S	S	S	S	S	S	S	**R**
Other agents	Chloramphenicol	S	S	S	S	S	S	S	S	S	S	S	S	S	S	S	S	S	S
	Trimethoprim/sulfamethoxazole	S	S	S	S	S	**R**	**R**	**R**	**R**	**R**	S	S	S	S	S	S	S	S

Legend: R, Resistant; I, Intermediate; S, Sensitive; F, Feces; W, Wastewater; L, Lagoon; SK, Soil. The first number is the number of the sample, and the second number is the number of the isolate.

**Table 4:** Antibiotic resistances from the disk diffusion method of some of the isolated *E. coli* strains from the farm near Veliko Turnovo (FV1.1–FV4.3).

Drug Class	Antibiotic/Strain	FV1.1	FV2.1	FV2.2	FV2.3	FV2.4	FV2.5	FV2.6	FV2.7	FV3.1	FV3.2	FV3.3	FV4.1	FV4.2	FV4.3
Tetracyclines	Tetracycline	**R**	**R**	**R**	**R**	**R**	I	**R**	I	**R**	**R**	**R**	I	**R**	**R**
	Doxycycline hydrochloride	**R**	**R**	**R**	I	I	**R**	I	I	I	**R**	I	I	**R**	**R**
Macrolides	Erythromycin	I	I	I	I	I	I	I	S	I	I	I	I	I	**R**
Cephalosporins	Cefamandole	S	S	S	S	S	S	S	S	S	S	S	S	S	S
Fluoroquinolones	Nalidixic acid	S	S	S	I	S	S	S	I	S	S	S	S	S	**R**
	Pefloxacin	S	S	I	**R**	S	S	I	**R**	S	S	S	S	**R**	**R**
	Ciprofloxacin	S	S	S	S	S	S	S	S	S	S	S	S	S	I
Penicillins	Ampicillin	**R**	**R**	S	**R**	**R**	**R**	**R**	**R**	S	S	S	S	**R**	**R**
	Amoxicillin	S	**R**	S	**R**	**R**	**R**	**R**	**R**	S	S	S	S	**R**	**R**
	Amoxicillin/clavulanic acid	S	S	S	S	S	S	S	S	S	S	S	**R**	**R**	S
	Carbenicillin	S	I	I	I	I	I	I	I	S	S	S	S	I	I
Carbapenems	Meropenem	S	S	S	S	S	S	S	S	S	S	S	S	I	S
Aminoglycosides	Streptomycin	S	S	S	**R**	S	S	S	S	I	I	**R**	**R**	I	**R**
Other agents	Chloramphenicol	S	S	**R**	S	S	**R**	**R**	**R**	S	S	S	S	S	**R**
	Trimethoprim/sulfamethoxazole	S	S	**R**	**R**	S	S	**R**	**R**	I	S	**R**	S	S	**R**

Legend: R, Resistant; I, Intermediate; S, Sensitive; F, Feces. The first number is the number of the sample, and the second number is the number of isolate.

**Table 5:** Antibiotic resistances from the disk diffusion method of some of the isolated *E. coli* strains from the farm near Veliko Turnovo (FV4.4–WV4.2).

Drug Class	Antibiotic/Strain	FV4.4	WV1.2	WV1.3	WV1.4	WV1.5	WV1.7	WV2.1	WV2.2	WV2.3	WV2.5	WV2.6	WV4.1	WV4.2
Tetracyclines	Tetracycline	**R**	**R**	**R**	**R**	I	S	I	S	**R**	**R**	S	**R**	**R**
	Doxycycline hydrochloride	**R**	**R**	**R**	**R**	S	S	I	S	S	**R**	S	**R**	**R**
Macrolides	Erythromycin	**R**	I	I	I	I	I	I	I	I	I	I	I	I
Cephalosporins	Cefamandole	S	S	S	S	S	S	S	S	S	S	S	S	S
Fluoroquinolones	Nalidixic acid	**R**	S	S	S	S	S	S	S	S	S	S	S	S
	Pefloxacin	**R**	I	I	I	S	S	S	I	S	**R**	I	S	S
	Ciprofloxacin	I	S	S	S	S	S	S	S	S	S	S	S	S
Penicillins	Ampicillin	**R**	**R**	**R**	**R**	**R**	S	S	S	S	**R**	S	**R**	S
	Amoxicillin	**R**	**R**	**R**	**R**	**R**	S	S	S	S	**R**	S	**R**	S
	Amoxicillin/clavulanic acid	**R**	S	S	S	S	S	S	S	S	S	S	**R**	S
	Carbenicillin	I	S	I	I	S	I	I	S	S	S	S	I	I
Carbapenems	Meropenem	S	S	I	S	S	I	S	S	S	S	I	S	S
Aminoglycosides	Streptomycin	**R**	S	S	S	S	S	S	S	S	S	S	I	I
Other agents	Chloramphenicol	**R**	S	S	S	S	S	S	S	S	**R**	S	S	S
	Trimethoprim/sulfamethoxazole	**R**	S	S	**R**	I	S	S	S	S	**R**	S	S	S

Legend: R, Resistant; I, Intermediate; S, Sensitive; F, Feces; W, Wastewater. The first number is the number of the sample, and the second number is the number of the isolate.

**Table 6:** Antibiotic resistances from the disk diffusion method of some of the isolated *E. coli* strains from the farm near Veliko Turnovo (WV4.3–SV3.4).

Drug Class	Antibiotic/Strain	WV4.3	WV4.4	WV4.5	WV4.6	LV1.1	LV1.2	LV1.3	LV1.5	LV1.6	SV3.1	SV3.2	SV3.3	SV3.4
Tetracyclines	Tetracycline	I	**R**	**R**	**R**	**R**	**R**	**R**	**R**	**R**	**R**	S	**R**	I
	Doxycycline hydrochloride	I	**R**	**R**	**R**	**R**	I	**R**	**R**	S	**R**	S	**R**	S
Macrolides	Erythromycin	I	I	I	I	I	I	I	I	I	I	I	I	I
Cephalosporins	Cefamandole	S	S	S	S	S	S	S	S	S	S	S	S	S
Fluoroquinolones	Nalidixic acid	S	S	I	S	S	S	S	S	S	S	S	S	S
	Pefloxacin	S	I	**R**	I	I	S	I	S	S	S	S	S	S
	Ciprofloxacin	S	S	S	S	S	S	S	S	S	S	S	S	S
Penicillins	Ampicillin	**R**	S	**R**	**R**	S	S	**R**	S	S	S	S	S	S
	Amoxicillin	**R**	S	**R**	**R**	S	S	**R**	S	S	S	S	S	S
	Amoxicillin/clavulanic acid	S	S	**R**	I	S	S	S	S	S	S	S	S	S
	Carbenicillin	S	S	**R**	S	I	S	I	S	S	S	I	S	I
Carbapenems	Meropenem	S	S	S	S	I	S	I	S	S	S	S	S	S
Aminoglycosides	Streptomycin	S	**R**	**R**	S	**R**	S	S	S	S	S	S	S	S
Other agents	Chloramphenicol	**R**	S	**R**	**R**	S	**R**	S	S	S	S	S	S	S
	Trimethoprim/sulfamethoxazole	S	S	**R**	S	S	**R**	S	S	S	S	S	S	S

Legend: R, Resistant; I, Intermediate; S, Sensitive; W, Wastewater; L, Lagoon; SV, Soil. The first number is the number of the sample, and the second number is the number of the isolate.

**Table 7:** Antibiotic resistances from the disk diffusion method of some of the isolated *E. coli* strains from the farm near Karnobat (F1.1–W2.4).

Drug Class	Antibiotic/Strain	F1.1	F2.1	F2.2	F3.1	F4.1	F4.2	F5.1	F6.1	F6.2	W1.1	W1.2	W2.1	W2.2	W2.3	W2.4
Tetracyclines	Tetracycline	**R**	**R**	**R**	**R**	**R**	**R**	**R**	**R**	**R**	**R**	I	S	**R**	**R**	**R**
	Doxycycline hydrochloride	**R**	**R**	**R**	**R**	**R**	**R**	**R**	**R**	**R**	**R**	I	**R**	S	**R**	**R**
Macrolides	Erythromycin	I	I	I	**R**	I	I	I	**R**	**R**	I	I	I	I	I	I
Cephalosporins	Cefamandole	S	S	S	S	S	S	I	S	S	S	S	S	S	S	S
Fluoroquinolones	Nalidixic acid	S	S	S	S	S	S	S	S	S	S	S	S	S	S	S
	Pefloxacin	S	S	S	S	S	S	S	S	S	S	S	S	**R**	S	S
	Ciprofloxacin	S	S	S	S	S	S	S	S	I	S	S	S	S	S	S
Penicillins	Ampicillin	S	**R**	**R**	S	**R**	**R**	**R**	**R**	**R**	**R**	S	**R**	S	**R**	S
	Amoxicillin	S	**R**	**R**	S	**R**	**R**	**R**	**R**	**R**	**R**	S	**R**	S	**R**	S
	Amoxicillin/clavulanic acid	**R**	S	**R**	S	**R**	**R**	**R**	**R**	**R**	S	S	S	S	S	S
	Carbenicillin	I	I	**R**	S	S	S	I	I	I	S	S	I	S	S	S
Carbapenems	Meropenem	S	S	S	S	S	S	S	S	S	S	S	S	S	S	S
Aminoglycosides	Streptomycin	**R**	**R**	**R**	**R**	**R**	**R**	**R**	S	I	S	S	**R**	I	I	**R**
Other agents	Chloramphenicol	**R**	**R**	**R**	**R**	S	S	**R**	**R**	**R**	**R**	**R**	**R**	**R**	**R**	S
	Trimethoprim/sulfamethoxazole	S	**R**	S	S	**R**	S	**R**	S	S	S	**R**	S	S	S	S

Legend: R, Resistant; I, Intermediate; S, Sensitive; F, Feces; W, Wastewater. The first number is the number of the sample, and the second number is the number of the isolate.

**Table 8:** Antibiotic resistances from the disk diffusion method of some of the isolated *E. coli* strains from the farm near Karnobat (F1.1–S7.2) and the controls.

Drug Class	Antibiotic/Strain	L1.1	L1.2	L2.1	L2.2	T1.1	T1.2	T1.3	S6.1	S6.2	S6.3	S7.2	*E. coli* O:157	*E. coli* ATCC 35218
Tetracyclines	Tetracycline	S	S	**R**	**R**	I	I	**R**	**R**	**R**	**R**	**R**	**R**	S
	Doxycycline hydrochloride	S	I	**R**	**R**	**R**	**R**	**R**	**R**	**R**	**R**	**R**	S	S
Macrolides	Erythromycin	I	S	I	I	I	I	I	I	I	I	I	S	S
Cephalosporins	Cefamandole	S	S	S	S	S	S	S	S	S	S	S	S	S
Fluoroquinolones	Nalidixic acid	S	S	S	S	**R**	S	S	S	S	I	S	S	**R**
	Pefloxacin	S	S	S	S	**R**	S	S	S	S	S	S	S	S
	Ciprofloxacin	S	S	S	S	**R**	S	S	S	S	S	S	S	S
Penicillins	Ampicillin	S	S	S	**R**	S	**R**	**R**	S	S	S	**R**	S	**R**
	Amoxicillin	S	S	S	**R**	S	**R**	**R**	S	S	S	**R**	-	**R**
	Amoxicillin/clavulanic acid	S	S	S	S	S	**R**	**R**	S	S	S	**R**	S	S
	Carbenicillin	S	S	S	S	I	S	S	S	S	I	S	S	S
Carbapenems	Meropenem	S	S	S	S	S	S	S	S	S	S	S	S	S
Aminoglycosides	Streptomycin	I	**R**	**R**	**R**	S	S	I	**R**	**R**	**R**	I	S	**R**
Other agents	Chloramphenicol	**R**	**R**	**R**	**R**	**R**	**R**	**R**	**R**	**R**	**R**	**R**	**R**	**R**
	Trimethoprim/sulfamethoxazole	S	S	S	S	**R**	S	**R**	S	S	**R**	S	S	S

Legend: R, Resistant; I, Intermediate; S, Sensitive; L, Lagoon; T, Transport vehicle; S6 and S7, Soil. The first number is the number of the sample, and the second number is the number of isolate.

**Table 9:** Adherence of isolated *E. coli* from the pig farm near Kostinbrod, compared with that of the controls.

Strain	OD_550 nm_	Adherence	Biofilm	Strain	OD_550 nm_	Adherence	Biofilm
ATCC 35218	0.676	SA	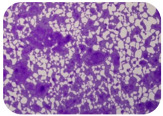	WK2.6	0.711	SA	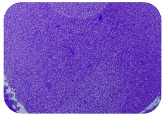
O157	0.321	MA	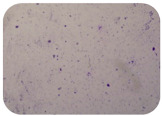	WK3.3	0.239	WA	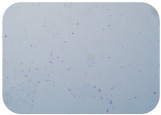
Blank	0.156	-	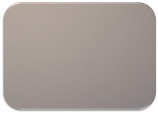	LK1.1	0.224	WA	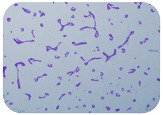
FK3.1	0.196	WA	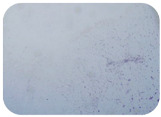	LK1.3	0.188	WA	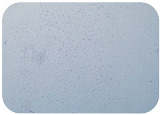
FK3.2	0.237	WA	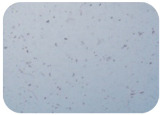	LK1.6	0.174	WA	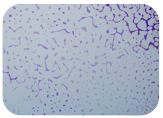
FK3.3	0.254	WA	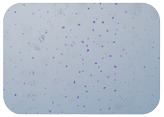	LK2.1	0.242	WA	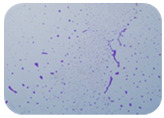
FK3.4	0.229	WA	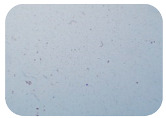	LK2.2	0.222	WA	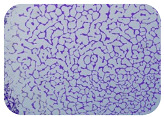
FK3.5	0.293	WA	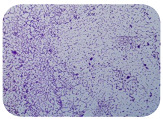	LK3.1	0.215	WA	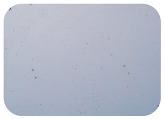
WK1.3	0.859	SA	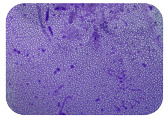	LK3.4	0.287	WA	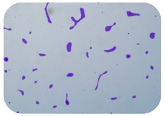
WK1.4	0.864	SA	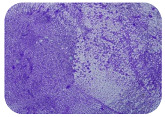	SK3.1	0.580	MA	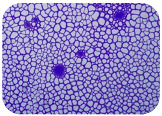
WK2.5	0.680	SA	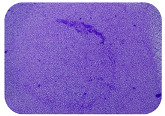				

**Table 10:** Adherence of a part of the isolated *E. coli* from the pig farm near Veliko Turnovo, compared with that of the controls.

Strain	OD_550 nm_	Adherence	Biofilm	Strain	OD_550 nm_	Adherence	Biofilm
ATCC 35218	0.676	SA	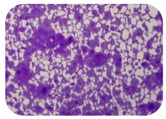	FV3.1	0.208	WA	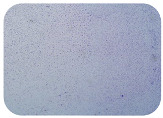
O157	0.321	MA	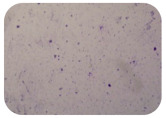	FV3.2	0.143	NA	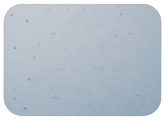
Blank	0.156	-	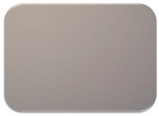	FV3.3	0.262	WA	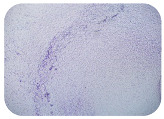
FV1.1	0.382	MA	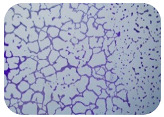	FV4.1	0.193	WA	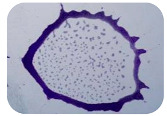
FV2.1	0.269	WA	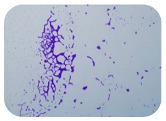	FV4.2	0.293	WA	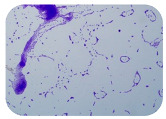
FV2.2	0.246	WA	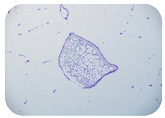	FV4.3	0.204	WA	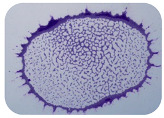
FV2.3	0.313	MA	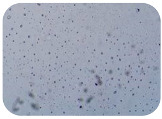	FV4.4	0.323	MA	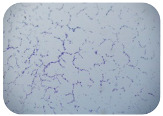
FV2.4	0.238	WA	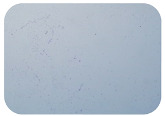	WV1.2	0.192	WA	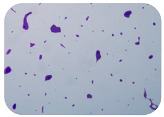
FV2.5	0.330	MA	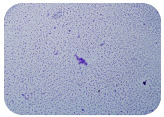	WV1.3	0.283	WA	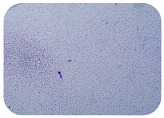
FV2.6	0.351	MA	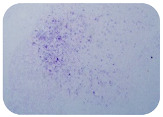	WV1.4	0.253	WA	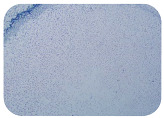
FV2.7	0.200	WA	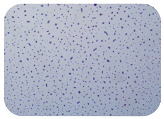	WV1.5	0.229	WA	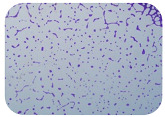

**Table 11:** Adherence of the other part of the isolated *E. coli* from the pig farm near Veliko Turnovo, compared with that of the controls.

Strain	OD_550 nm_	Adherence	Biofilm	Strain	OD_550 nm_	Adherence	Biofilm
ATCC 35218	0.676	SA	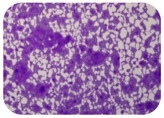	WV4.4	0.275	WA	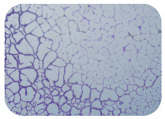
O157	0.321	MA	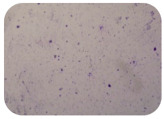	WVT4.5	0.231	WA	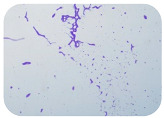
Blank	0.156	-	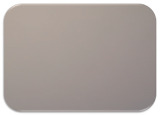	WV4.6	0.264	WA	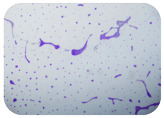
WV1.7	0.168	WA	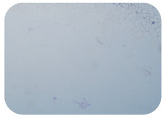	LV1.1	0.300	WA	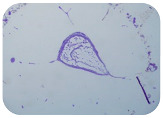
WV2.1	0.499	MA	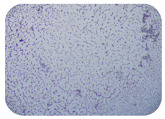	LV1.2	0.442	MA	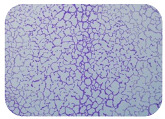
WV2.2	0.314	MA	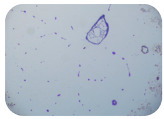	LV1.3	0.202	WA	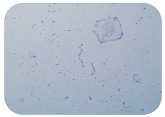
WV2.3	0.302	WA	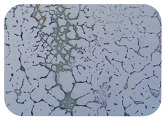	LV1.5	0.232	WA	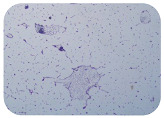
WV2.5	0.175	WA	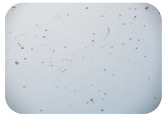	LV1.6	0.261	WA	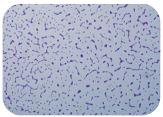
WV2.6	0.258	WA	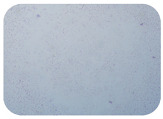	SV3.1	0.550	MA	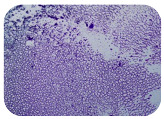
WV4.1	0.207	WA	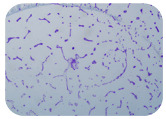	SV3.2	0.747	SA	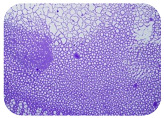
WV4.2	0.192	WA	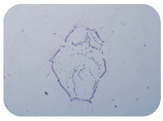	SV3.3	0.996	MA	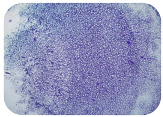
WV4.3	0.406	MA	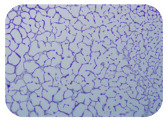	SV3.4	0.780	SA	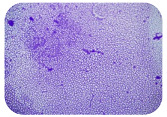

**Table 12:** Adherence of the isolated *E. coli* from the pig farm near Karnobat, compared with that of the controls.

Strain	OD_550 nm_	Adherence	Biofilm	Strain	OD_550 nm_	Adherence	Biofilm
ATCC 35218	0.680	SA	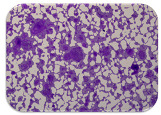	W2.2	0.645	SA	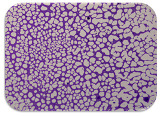
O157	0.317	MA	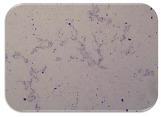	W2.3	0.975	SA	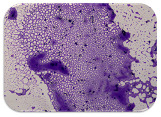
Blank	0.156	-	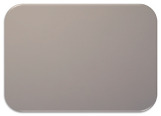	W2.4	0.532	MA	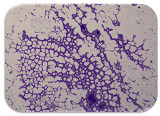
F1.1	0.469	MA	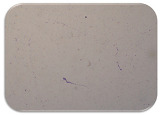	L1.1	0.737	SA	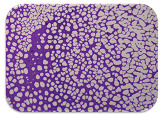
F2.1	0.461	MA	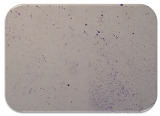	L1.2	0.759	SA	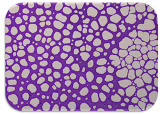
F2.2	0.235	WA	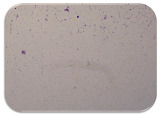	L2.1	1.084	SA	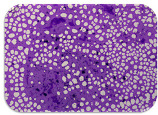
F3.1	0.278	WA	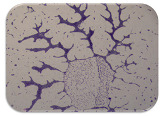	L2.2	0.190	WA	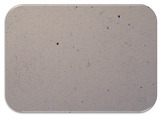
F4.1	0.229	WA	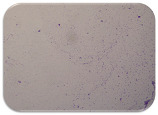	T1.1	0.423	MA	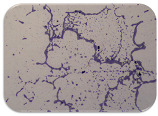
F4.2	0.323	MA	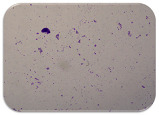	T1.2	0.461	MA	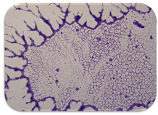
F5.1	0.306	WA	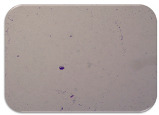	T1.3	0.502	MA	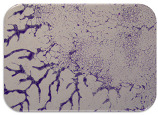
F6.1	0.650	SA	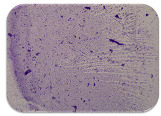	S6.1	0.266	WA	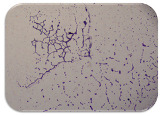
F6.2	0.686	SA	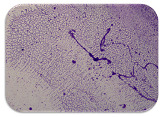	S6.2	0.295	WA	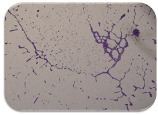
W1.1	0.317	MA	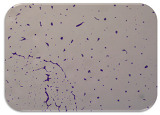	S6.3	0.455	MA	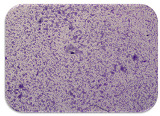
W1.2	0.568	MA	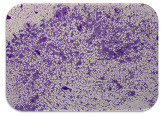	S7.2	0.546	MA	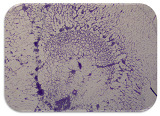
W2.1	0.949	SA	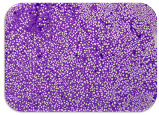				

Legend: NA, non-adherent confirmed *E. coli* strains; WA, weakly adherent confirmed *E. coli* strains; MA, moderately adherent confirmed *E. coli* strains; SA, strongly adherent confirmed *E. coli* strains.

## Data Availability

The data presented in this study are available on request from the corresponding author.
